# ESR1 Gene Mutation in Hormone Receptor-Positive HER2-Negative Metastatic Breast Cancer Patients: Concordance Between Tumor Tissue and Circulating Tumor DNA Analysis

**DOI:** 10.3389/fonc.2021.625636

**Published:** 2021-03-11

**Authors:** Loredana Urso, Grazia Vernaci, Jessica Carlet, Marcello Lo Mele, Matteo Fassan, Elisabetta Zulato, Giovanni Faggioni, Alice Menichetti, Elisabetta Di Liso, Gaia Griguolo, Cristina Falci, Pierfranco Conte, Stefano Indraccolo, Valentina Guarneri, Maria Vittoria Dieci

**Affiliations:** ^1^ Department of Surgery, Oncology and Gastroenterology—DiSCOG, University of Padova, Padova, Italy; ^2^ Medical Oncology 2, Istituto Oncologico Veneto IOV-IRCCS, Padova, Italy; ^3^ Department of Pathology, Azienda Ospedaliera Universitaria, Padova, Italy; ^4^ Department of Medicine-DIMED, Surgical Pathology and Cytopathology Unit, University of Padua, Padova, Italy; ^5^ Immunology and Molecular Oncology Unit, Istituto Oncologico Veneto IOV-IRCCS, Padova, Italy

**Keywords:** Estrogen Receptor 1 (ESR1), metastatic breast cancer, endocrine therapy, ctDNA, liquid biopsy

## Abstract

Endocrine therapy represents the cornerstone of treatment in hormone receptor-positive (HR+), HER2-negative metastatic breast cancer (mBC). The natural course of this disease is marked by endocrine resistance, mainly due to Estrogen Receptor 1 (ESR1) acquired mutations. The aim of this study is to evaluate the concordance between ESR1 status in metastatic tumor specimens and matched circulating tumor DNA (ctDNA). Forty-three patients with HR+, HER2-negative mBC underwent both a metastatic tumor biopsy and a liquid biopsy at the time of disease progression. DNA extracted from formalin fixed paraffin embedded (FFPE) tumor specimens and ctDNA from matched plasma were analyzed by droplet digital (dd)PCR for the main ESR1 mutations (Y537S, Y537C, Y537N, D538G, E380Q). We observed a total mutation rate of 21%. We found six mutations on tissue biopsy: Y537S (1), D538G (2), Y537N (1), E380Q (2). Three patients with no mutations in tumor tissue had mutations detected in ctDNA. The total concordance rate between ESR1 status on tumor tissue and plasma was 91%. Our results confirm the potential role of liquid biopsy as a non-invasive alternative to tissue biopsy for ESR1 mutation assessment in mBC patients.

## Introduction

Hormone receptor-positive (HR+) breast cancer (BC) accounts for about one third of all BC (1). Endocrine manipulation is the mainstay of treatment of HR+/human epidermal growth factor 2- negative (HER2-) BC, and the traditional armamentarium includes aromatase inhibitors (AI), selective estrogen receptor modulators (SERMs, as tamoxifen), selective estrogen receptor degraders (SERDs, as fulvestrant).

However, in the metastatic setting, development of resistance invariably occurs, and about 15–20% of patients show *de novo* resistance (2–4). Several mechanisms have been linked to endocrine resistance, including mutation in Estrogen Receptor 1 (*ESR1*) gene. This gene, located on chromosome 6, encodes for ER*α*, a member of the nuclear hormone receptors superfamily (5). In response to estrogens, ER interacts with specific estrogen response elements (EREs) on DNA and promotes cell proliferation. Moreover, ER harbors numerous bi-directional cross-talks with membrane tyrosine kinase receptors such as epidermal growth factor (EGFR), HER2, insulin-like growth factor (IGFR), that play an important role in breast cancer cells’ growth and survival (6–9).

*ESR1* mutations mostly occur in specific hotspots located in the ligand-binding domain of the receptor and result in estrogen-independent function of ER (10). The most common ESR1 mutations are Y537S/N/C and D538G (11).

*ESR1* mutations are rare in primary BC and become more frequent in the metastatic setting, with a total rate of about 30% (12, 13). These mutations are relatively rare in patients treated with tamoxifen only and typically develop after previous exposure to aromatase inhibitors, as a result of the selective pressure of endocrine deprivation therapies (12–18).

Mutant cells are resistant to AI *in vitro*, while high doses of tamoxifen and fulvestrant inhibit signaling of mutant ER (14, 19). In the combined analysis of the SoFEA and EFECT trials, ESR1 mutations have been shown to be associated with worse progression-free survival (PFS) and overall survival (OS) in patients treated with exemestane *versus* fulvestrant, with an objective response rate of 9.5 *versus* 0.0% on respectively fulvestrant and exemestane (17, 20). These findings confirm that ESR1 mutated patients still derive clinical benefit from endocrine therapy with fulvestrant. In this context, preclinical data have shown the effectiveness of new potent oral SERDs (21–24).

Whether detection of ESR1 mutation could impact on treatment decision is still under investigation (NCT03079011).

Circulating tumor DNA (ctDNA) is a cell-free DNA released by tumor cells in the blood (25). ctDNA can be detected in the plasma of patients with cancer, and its analysis may represent a non-invasive tool for detecting and monitoring key gene mutations.

Although different studies showed the potential of Next Generation sequencing (NGS) or Droplet Digital PCR (ddPCR) analyses in identifying ESR1 mutations in ctDNA from HR+ metastatic breast cancer (mBC), few reports compared the sensitivity of the detection in tissue specimens compared to matched plasma samples (26–30).

We conducted a prospective study in a cohort of HR+/HER2- mBC patients to assess the concordance of ESR1 mutation evaluated on matched tumor tissue samples from a metastatic lesion and ctDNA from plasma.

## Materials and Methods

### Patients

The study population was represented by a prospective cohort of 43 HR+/HER2- (defined as ER or PgR expression ≥10% and HER2 immunohistochemical 0**–**1+ or 2+ with no amplification at fluorescence *in situ* hybridization) mBC patients (Age ≥ 18 years) who underwent a biopsy of a metastatic lesion at our Institution, as part of the routine diagnostic-therapeutic management, prior to the start of a new line of systemic treatment. Patients were enrolled from July 2018 to August 2020.

Patients were registered in a prospective database reporting demographics, clinical-pathological features, type of treatment for early (eBC) and advanced BC (aBC), results for ESR1 mutation, and follow-up data.

Treatment for metastatic disease was administered in accordance to national guidelines.

The study (SPIDER) was approved by the Ethic Committee of Istituto Oncologico Veneto (Cod. CESC IOV 2018/26, February 26, 2018). Informed consent was obtained from all subjects.

### Samples Preparation and Analysis

#### DNA Extraction From Formalin-Fixed Paraffin-Embedded Tissue Biopsy

We collected 43 FFPE tumor biopsies, reviewed by a pathologist (MF) for tumor tissue quality and quantity. Genomic DNA (gDNA) was extracted from five FFPE sections containing at least 30% of tumor cells using QIAmp^®^ DNA Micro Kit (Qiagen) following the manufacturer’s instructions. DNA was quantified by Nanodrop One (Thermo Scientific^®^). Twenty ng of total gDNA was used for the detection of ESR1 mutations.

#### Plasma Sample Collection and DNA Extraction

Liquid biopsy was performed at the same time point of tissue collection, simultaneously with the routine blood exams, with no additional venipuncture. Twenty ml of blood samples was collected in two Helix ctDNA Stabilization tubes (Diatech Pharmacogenetics SRL) and processed within 24 h. Plasma was separated by centrifugation at 2,000 × g for 10 min at 4°C. Next, to further purify plasma from corpuscular cells, the supernatant was centrifuged at 20,000 × g for 10 min at 4°C. Plasma was stored at −80°C until analysis. ctDNA was extracted from 2 ml of plasma using the Maxwell^®^ RSC ccfDNA Plasma Kit (Promega, Madison, Wisconsin, USA), and quantified using Qubit dsDNA HS Assay Kit (Life Technologies, USA). 7.5 µl of ctDNA was used for the detection of ESR1 mutations.

### Detection of ESR1 Mutations by ddPCR

ESR1 mutations were analyzed by ddPCR on the QX200 ddPCR system (Bio-Rad Laboratories) following the manufacturer’s instructions. We assessed the following hotspots in 43 tissue specimens: Y537S, Y537C, Y537N, D538G, E380Q. These hotspots were selected based on their frequency among ESR1 mutations in published studies (11) and in the COSMIC dataset (v92). ddPCR probes were purchased from Bio-Rad. To set-up the method, each assay was tested using pSEPT plasmid bearing the indicated mutations. All samples had adequate proportion of tumor cells. All the mutations detected in tumor tissue biopsy were checked in matched ctDNA. As Y537C/N was barely detected in our tissue sample cohort (0.0 and 2.3%, respectively), all plasma samples were analyzed for the three main hotspots: Y537S, D538G, E380Q. Each sample of tumor tissue DNA was run in duplicate; each sample of cfDNA was run in triplicate.

We defined a positive mutation in tissue DNA with a threshold of 1% allele frequency to avoid technical biases from fixation process (31); for ctDNA we used a cutoff of three mutant-positive droplets per well, following the manufacturer guidelines. Allele frequency for each mutation was determined considering fractional abundance of mutated droplets above the total.

### Sample Size and Statistical Analysis

Statistical analyses were conducted using IBM SPSS (Version 20) Software. The association between categorical variables was evaluated using the χ2 test.

We evaluated the concordance between ESR1 mutation analysis on matched tissue DNA and ctDNA samples. We considered three concordance measures: i) the rate of ctDNA mutated samples over the total of tumor tissue mutated samples (ctDNA confirmation rate), ii) the rate of concordant mutated matched pairs over the total of pairs showing at least one mutated sample (ctDNA, tumor tissue, or both; concordance mutation rate), and iii) the rate of concordant mutated or concordant wild-type matched pairs over the total of 43 analyzed pairs (total concordance rate).

PFS was calculated as the time interval from the date of liquid biopsy to disease progression or death, whichever was first. OS was calculated from the date of liquid biopsy to death. Patients without an event were censored at the date of last follow-up.

Survival curves were estimated using the Kaplan**–**Meier model and we used the log-rank test to study differences between groups. For all the performed tests, significance was inferred for a value p <0.05.

## Results

### Patients’ Characteristics

From July 2018 to August 2020 we enrolled 43 patients.

Clinicopathologic characteristics at the time of first breast cancer diagnosis are reported in **Table 1**. Seventeen patients had a stage IV *de novo* disease at the time of first diagnosis. All patients had HR+/HER2- tumor phenotype as defined by the protocol on at least one tumor biopsy (either primary tumor or relapse). Among those patients who experienced a disease relapse after a prior diagnosis of primary breast cancer, all but three had a concordant HR-positive and HER2-negative tumor phenotype on both primary tumor and relapse biopsy. Three patients with HER2-positive (n = 2) and triple negative (n = 1) primary breast cancer had a subsequent relapse biopsy showing HR-positive and HER2-negative tumor phenotype. For one patient with HR+/HER2− phenotype on relapse biopsy, receptor status of the primary tumor was not available (**Supplementary Tables 2** **if** **3**). Median age at first breast cancer diagnosis was 50 years (range 42**–**62). Most patients had a tumor of ductal histology (n = 37, 86%) and histologic grade 3 (n = 24, 56%). Treatments for early breast cancer are listed in **Table 1**.

**Table 1:** Clinical-pathological characteristics at diagnosis and treatment for eBC.

			N (%)	
Age (median)			50 (42-62)	
Menopause	Yes		25 (58)	
	No		18 (42)	
Tumor histotype	Ductal		37 (86)	
	Lobular		4 (9)	
	Other		2 (5)	
Estrogen receptor	Positive		39 (91)	
	Negative		3 (7)	
	NA		1 (2)	
Progesteron receptor	Positive		34 (79)	
	Negative		8 (19)	
	NA		1 (2)	
Histologic Grade	1		1 (2)	
	2		15 (35)	
	3		24 (56)	
	NA		3 (7)	
HER2	Positive		2 (5)	
	Negative		39 (90)	
	NA		2 (5)	
Stage (AJCC)	I		7 (16)	
	II		9 (21)	
	III		9 (21)	
	IV		17 (40)	
	NA		1 (2)	
CT for eBC	Yes		23 (53)	
	No		20 (47)	
HT for eBC	Yes		22 (51)	
		Tam		5 (23)
		AI		9 (41)
		Tam + AI		7 (32)
		NA		1 (4)
	No		21 (49)	

N, number of patients; HER2, human epidermal growth factor receptor 2; CT, chemotherapy; HT, hormone therapy; eBC, early breast cancer.

Patients’ characteristics at the time of enrollment in this study are reported in **Table 2**. The majority of patients presented with visceral metastases (n = 29, 67%), and half of the patients had more than three metastatic sites involved (n = 22, 51%) (**Supplementary Table 4**). Twenty-eight patients (65%) had not received any prior systemic therapy for advanced disease at the time of enrolment; 26 patients had been previously exposed to AIs for the treatment of early and/or advanced disease (60%).

**Table 2:** Patients’ characteristics according to ESR1 status.

		ESR1 (tissue and/or ctDNA)			
		Total N (%)	WT N (%)	Mut N (%)	P value
Visceral metastasis	Yes	29 (67)	21 (62)	8 (89)	0.123
	No	14 (33)	13 (38)	1 (11)	
N° metastatic sites	<3	21 (49)	16 (47)	5 (56)	0.650
	≥3	22 (51)	18 (53)	4 (44)	
LDH	Low	1 (4)	1 (5)	0	0.091
	Normal	13 (54)	13 (62)	0	
	High	10 (42)	7 (33)	3 (100)	
Previous systemic therapies for advanced disease	0	28 (65)	25 (73)	3 (33)	0.77
	1–2	7 (16)	4 (12)	3 (33)	
	≥3	8 (19)	5 (15)	3 (34)	
Prior exposure to AI (eBC and/or aBC)	Yes	26 (62)	17 (52)	9 (100)	**0.010**
	No	16 (38)	16 (48)	0	
Prior exposure to CT (eBC and/or aBC)	Yes	11 (26)	7 (21)	4 (44)	0.160
	No	32 (74)	27 (79)	5 (56)	
Prior exposure to Tam only (eBC and/or aBC)	Yes	5 (24)	3 (21)	2 (29)	0.717
	No	16 (76)	11 (79)	5 (71)	
Prior exposure to AI for aBC	Yes	13 (30)	7 (21)	6 (67)	**0.008**
	No	30 (70)	27 (79)	3 (33)	
Prior exposure to Fulvestrant for aBC	Yes	9 (21)	4 (12)	5 (56)	**0.004**
	No	34 (79)	30 (88)	4 (44)	
Prior exposure to CDK 4/6 inh for aBC	Yes	9 (21)	5 (15)	4 (44)	0.051
	No	34 (79)	29 (85)	5 (56)	
Prior exposure to everolimus for aBC	Yes	5 (12)	2 (6)	3 (33)	**0.022**
	No	38 (88)	32 (94)	6 (67)	

Bold values are statistically significant. N, number of patients; WT, wild-type; Mut, mutant; LDH, lactate dehydrogenase; AI, aromatase inhibitor; eBC, early Breast Cancer; aBC, advanced Breast Cancer; CT, chemotherapy; Tam, tamoxifen; CDK, cyclin-dependent kinase. Bold values are statistically significant.

### Concordance of ESR1 Mutation on Tumor Tissue Biopsies and ctDNA

We identified the following ESR1 mutations in six of 43 patients (14%) on DNA extracted from tumor biopsies: Y537S (one subject), Y537N (one subject) E380Q (two subjects), D538G (two subjects). Four of the six mutations were confirmed on ctDNA (ctDNA confirmation rate: 67%) (**Supplementary Figure 1**). The low concentration of cfDNA in the two discordant cases, with less than 700 total droplets detected in the plasma, probably reduced the sensitivity of the test.

In order to verify that identified mutations were acquired *de novo*, we tested four out of six matched primary tumors, and no mutations were found (data not shown).

The most frequent ESR1 mutations (Y537S, D538G, and E380Q) were assessed on all ctDNA samples. For three WT tissue samples, the analysis of matched plasma revealed the D538G mutation in ctDNA, one of these ctDNA samples showed two concomitant hotspot mutations (D538G and Y537S). Two of these three patients with ESR1 mutation detected on ctDNA and not on tumor tissue had a high disease burden, with more than three metastatic sites and visceral involvement.

**Figure 1:**
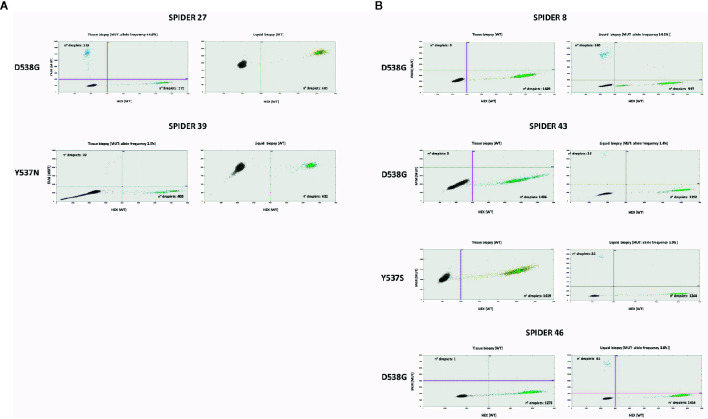
Discordant cases in ddPCR between tissue and liquid biopsy: ddPCR plots show the number of positive droplets for the indicated hotspot mutations (FAM+: blue dots), and the number of wt droplets (HEX+: green dots). Double positive droplets (FAM+/HEX+) were excluded from the analysis. **(A)** Samples mutated in tissue biopsy and WT in liquid biopsy. **(B)** Samples WT in tissue biopsy and mutated in liquid biopsy.

**Figure 1** shows the results of ddPCR for the discordant cases between tissue and plasma samples.

Our total ESR1 mutation rate, considering cases showing a mutation on tumor tissue and/or ctDNA over the total, was 21% (9/43).

**Figure 2:**
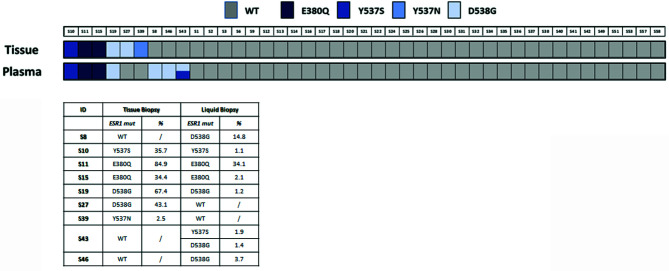
ESR1 mutation in tissue and plasma samples. The lower panel shows the specific ESR1 mutations found and their representation in samples as percentage (%) of mutated allele.

The concordance rate for mutation was 44% (four cases with ESR1 mutation on matched tissue and ctDNA over nine cases with a mutation detected on tissue and/or ctDNA samples). The total concordance rate (considering the Y537S, D538G and E380Q) between tumor tissue and plasma was 91% (39 concordant mutated or concordant wild-type matched pairs over 43 total pairs analyzed, **Figure 2** and **Supplementary Table 1**).

### Association of ESR1 Mutations With Patients’ Characteristics and Previous Therapies

**Table 2** shows the association of ESR1 mutation status with clinicopathological characteristics at study entry.

We found a statistically significant association between prior exposure to AI (considering both the early and the advanced setting and the advanced setting alone) and the presence of ESR1 mutation (p = 0.010 and p = 0.008 respectively). Prior exposure to fulvestrant and everolimus for aBC was also associated with higher rate of ESR1 mutation (p = 0.004 and p = 0.022 respectively). However, when we performed logistic regression multivariable analysis, none of these factors remained statistically significant after adjusting for the other variables (prior exposure to chemotherapy, aromatase inhibitors, tamoxifen, fulvestrant, CDK4/6 inhibitors, everolimus) (data not shown). The majority of the patients (n = 35, 81%) had received two or less previous treatment for BC, with no differences in terms of ESR1 mutational status. Features related with disease burden were not associated with presence of ESR1 mutation. Notably, we found a numerically higher rate of ESR1 mutation in the case of visceral disease (eight out nine mutated patients, p = 0.123). LDH value as assessed at the time of liquid biopsy was available for 24 patients. All of the three patients with ESR1 mutation had high LDH, although this association was not statistically significant (p = 0.091).

### Survival Analysis

Median follow-up was 14.5 months (95%CI 12.0–17.0 months).

**Figure 3:**
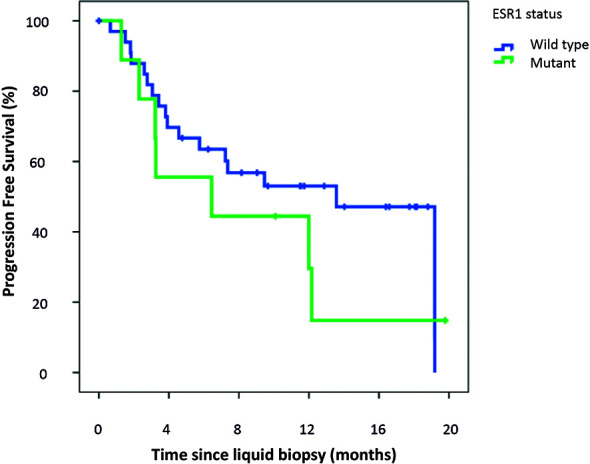
Kaplan–Meier progression-free survival analysis according to ESR1 status.

As shown in **Figure 3**, there was no significant difference in PFS between ESR1 wild-type and ESR1-mutated patients: median PFS was 13.6 months (95%CI 9.6–17.5 months) in ESR1 wild type population *versus* 6.4 months (95%CI 0.00–15.6 months) in ESR1 mutant patients (log-rank p = 0.283, HR 1.62, 95%CI 0.7–4.0, p = 0.288).

## Discussion

During the natural history of HR+/HER2-negative mBC, the onset of endocrine resistance is the rule, and a deep understanding of underlying mechanisms remains an unmet medical need.

Analysis of tumor tissue allows obtaining crucial predictive and prognostic information to guide clinicians, although, due to its static nature, a tumor biopsy is not able to capture intra-tumor heterogeneity and temporal evolution under exposure to specific treatments. Furthermore, multiple-biopsy testing could affect patients’ quality of life (QoL). In this perspective, liquid biopsy offers a charming tool to overcome these limitations.

In our work, we prospectively examined ESR1 status in the tissue of 43 patients with HR+/HER2- mBC and in matched plasma samples. Overall, our total mutation rate was 21%, consistent with main literature data (12, 14, 15, 26, 29).

In this study, the total concordance rate between ESR1 status on tumor tissue DNA and ctDNA was 91%. Among the five discordant cases described in our cohort, two out of six mutations detected in tumor tissue DNA were undetectable in ctDNA. A possible reason may be represented by the low concentration of cfDNA in these two patients. The other three discordant cases showed a mutation on ctDNA but not on matched tumor tissue DNA. This finding suggests that ctDNA might be able to represent the heterogeneity of mBC, particularly in the case of patients with multi-metastatic disease.

Available literature reports an overall concordance rate for ESR1 mutation between matched tissue and plasma samples ranging from 47 to 100%, although the majority of the data come from small series (**Table 3**) (15, 29, 30, 32, 36).

**Table 3:** Studies investigating concordance of ESR1 mutation between tissue and plasma.

Reference	Method	Total Mutation Rate	Overall concordance rate (%)*	Positive concordance rate (%)**
Schiavon G. (15)	ddPCR	19/171 (11.1%)^§^	30/31 (97%)	3/4 (75%)
Chu D. (30)	NGS	9/11 (82%) 3/8 (37.5%)^§^	10/11 (91%) 2/5 (40%)	8/9 (89%) 0/5 (0%)
Yanagawa T. (32)	NGS	11.3%	5/5 (100%)	0/5 (0%)
Sefrioui D. (33)	SANGER/ ddPCR	6/7 (86%)	5/7 (71.4%)	4/6 (67%)
Takeshita T. (29)	ddPCR	10/35(29%)	26/35 (74%)	1/10 (10%)
Lupini L. (34)	COLDPCR	8/40 (20%)	3/6 (50%)	1/4 (25%)
Spoerke J. (35)	rtPCR/ BEAMing	37.2%^§^	22/47 (47%)	11/36 (31%)
Wang P. (36)	ddPCR	3/43(7%)^†^ 4/35 (11.4%)	3/5 (60%)	3/5 (60%)

*Considering cases that were analyzed in both tissue and plasma samples and were both negative or both mutated.

**Considering cases that were positive for ESR1 mutation in tissue DNA and/or ctDNA and were positive in both tissue and ctDNA.

^§^prospective cohort.

^†^primary tumor.

ddPCR, droplet digital polymerase chain reaction; NGS, next generation sequencing.

Altogether, our results are consistent with previous studies which evaluated ESR1 mutation by ddPCR and showed a rate of concordance of 74 to 97% (15, 29).

Across available data, concordance rates appear to be lower when the ESR1 mutation status on ctDNA is compared with sequencing results obtained from archival tumor tissue samples rather than recent tumor biopsies, as performed in our study.

All patients with a detectable *ESR1* mutation (in either tissue DNA or ctDNA) had a previous exposure to AI (p = 0.010), confirming the role of the selective pressure of hormonal-deprivation therapy in endocrine resistance development (17, 35). With regard to other therapies for advanced disease, a statistically significant association between ESR1 mutation and fulvestrant (p = 0.004) and everolimus (p = 0.022) was evident. It must be taken into account that all these patients received AI either previously (in case of fulvestrant) or concomitantly (in case of everolimus). Thus, the exposure to such therapies is a surrogate of prior treatment with aromatase inhibitor.

In a recent meta-analysis, the presence of ESR1 mutation was associated with worse PFS and OS in a population of patients with HR+/HER2-negative mBC (37).

In our population, the presence of ESR1 mutation did not impact on either PFS or OS, although this finding could be biased by the small sample size with limited follow-up. Moreover, the limited sample size did not allow conducting a survival analysis stratified by line of treatment.

Although this study has limitations, such as the small sample size, the mono-institutional enrollment, and ESR1 status assessment in a single laboratory, our results showed high concordance rate between tumor tissue and ctDNA (91%), providing evidence of reliability and feasibility of liquid biopsy to analyze ESR1 mutation in breast cancer patients. Moreover, the presence of ESR1 mutation in ctDNA of three patients lacking ESR1 mutations in the tissue suggests that liquid biopsy may capture the heterogeneous genetic landscape of metastatic tumors.

In conclusion, our data confirm the potential role of liquid biopsy as a valid and preferable non-invasive alternative to tissue biopsy for ESR1 mutation assessment in mBC patients. Moreover, it can also allow longitudinal tracking of ESR1 mutations during the disease course at multiple timepoints without exposing patients to the risks related to invasive procedures. Clinical utility of this approach to guide treatment choices is currently under investigation.

## Data Availability Statement

The raw data supporting the conclusions of this article will be made available by the authors, without undue reservation.

## Ethics Statement

The studies involving human participants were reviewed and approved by IOV Ethics Committee (Cod. CESC IOV 2018/26, February 26, 2018). The patients/participants provided their written informed consent to participate in this study.

## Author Contributions

MVD conceived and designed the study. LU, JC, MLM, MF, and EZ performed molecular and pathology analyses. GV, GF, AM, EL, GG, CF, PC, VG, and MVD followed the patients. GV and LU acquired, analyzed, and interpretated data and drafted the work. MVD, VG, PC, and SI revised the work critically and approved the final version. All authors agree to be personally accountable for the content of the work. All authors contributed to the article and approved the submitted version.

## Funding

This research was supported by grant from Italian Ministry of Health (GR-2016-02361279 to MVD).

## Conflict of Interest

MVD reports personal fees for consultancy/advisory role from: Eli Lilly, Novartis, Celgene, Genomic Health, outside the submitted work.

The remaining authors declare that the research was conducted in the absence of any commercial or financial relationships that could be construed as a potential conflict of interest.
