# Supplementary material for: ESR1 Gene Mutation in Hormone Receptor-Positive HER2-Negative Metastatic Breast Cancer Patients: Concordance Between Tumor Tissue and Circulating Tumor DNA Analysis
Source: Front Oncol. 2021 Mar 11;11:625636. doi: 10.3389/fonc.2021.625636 (PMC7991720; doi:10.3389/fonc.2021.625636)
Supplement: Supplementary file 1 [file Table_1.docx]

Supplementary Table 1. Concordance of ESR1 status between tissue and ctDNA.

|  |  | ESR1 tissue | |
| --- | --- | --- | --- |
|  |  | Mutant | Wild type |
| ESR1 ctDNA | Mutant | 4 | 3 |
|  | Wild type | 2 | 34 |
