# Supplementary material for: ESR1 Gene Mutation in Hormone Receptor-Positive HER2-Negative Metastatic Breast Cancer Patients: Concordance Between Tumor Tissue and Circulating Tumor DNA Analysis
Source: Front Oncol. 2021 Mar 11;11:625636. doi: 10.3389/fonc.2021.625636 (PMC7991720; doi:10.3389/fonc.2021.625636)
Supplement: Supplementary file 2 [file Table_2.docx]

|  | **ER (%)** | **PR (%)** |
| --- | --- | --- |
| **S1** | 60 | 0 |
| **S2** | 1 | 0 |
| **S3** | 95 | 5 |
| **S6** | 80 | 0 |
| **S8** | 100 | 35 |
| **S9** | 100 | 5 |
| **S10** | 90 | 90 |
| **S11** | 70 | 40 |
| **S12** | 90 | 25 |
| **S13** | 95 | 95 |
| **S14** | 99 | 75 |
| **S15** | 90 | 70 |
| **S16** | 90 | 80 |
| **S17** | 100 | 15 |
| **S18** | 100 | 100 |
| **S19** | 100 | 100 |
| **S20** | 20 | 0 |
| **S21** | 100 | 98 |
| **S22** | 90 | 70 |
| **S24** | 90 | 0 |
| **S25** | 90 | 90 |
| **S26** | 88 | 30 |
| **S27** | 90 | 90 |
| **S28** | 35 | 3 |
| **S30** | 70 | 0 |
| **S31** | 100 | 0 |
| **S32** | 95 | 100 |
| **S34** | 80 | 22 |
| **S35** | 100 | 0 |
| **S36** | 100 | 35 |
| **S37** | 50 | 0 |
| **S38** | 100 | 0 |
| **S39** | 100 | 30 |
| **S40** | 80 | 0 |
| **S41** | 90 | 5 |
| **S42** | 70 | 40 |
| **S43** | 90 | 0 |
| **S46** | 90 | 0 |
| **S49** | 100 | 0 |
| **S51** | 95 | 30 |
| **S53** | 90 | 0 |
| **S57** | 90 | 40 |
| **S58** | 90 | 40 |

**Supplementary Table 2.** Hormone receptors expression of the 43 metastatic HER2 negative samples.
