# Supplementary material for: ESR1 Gene Mutation in Hormone Receptor-Positive HER2-Negative Metastatic Breast Cancer Patients: Concordance Between Tumor Tissue and Circulating Tumor DNA Analysis
Source: Front Oncol. 2021 Mar 11;11:625636. doi: 10.3389/fonc.2021.625636 (PMC7991720; doi:10.3389/fonc.2021.625636)
Supplement: Supplementary file 3 [file Table_3.docx]

**Supplementary Table 3.** Hormone receptors and HER2 expression of the 43 primary tumors.

|  | **ER (%)** | **PR (%)** | **HER2** |
| --- | --- | --- | --- |
| **S1** | 0 | 0 | negative |
| **S2** | 1 | 1 | positive |
| **S3** | 90 | 30 | negative |
| **S6** | 60 | 60 | negative |
| **S8** | 62 | 70 | positive |
| **S9** | 100 | 5 | negative |
| **S10** | 95 | 75 | negative |
| **S11** | 90 | 20 | negative |
| **S12** | 100 | 90 | negative |
| **S13** | 100 | 20 | negative |
| **S14** | 0 | 0 | NA |
| **S15** | 90 | 90 | negative |
| **S16** | 10 | 70 | negative |
| **S17** | 70 | 70 | negative |
| **S18** | 90 | 95 | negative |
| **S19** | 90 | 80 | negative |
| **S20** | 90 | 90 | negative |
| **S21** | 100 | 98 | negative |
| **S22** | 90 | 70 | negative |
| **S24** | 80 | 30 | negative |
| **S25** | 90 | 90 | negative |
| **S26** | 88 | 30 | negative |
| **S27** | 90 | 80 | negative |
| **S28** | 90 | 80 | negative |
| **S30** | 60 | 0 | negative |
| **S31** | 100 | 0 | negative |
| **S32** | 95 | 100 | negative |
| **S34** | 80 | 22 | negative |
| **S35** | 90 | 35 | negative |
| **S36** | 100 | 35 | negative |
| **S37** | 90 | 25 | negative |
| **S38** | 90 | 5 | negative |
| **S39** | 90 | 25 | negative |
| **S40** | 30 | 0 | negative |
| **S41** | 0 | 50 | negative |
| **S42** | 90 | 90 | negative |
| **S43** | 90 | 0 | negative |
| **S46** | 90 | 28 | negative |
| **S49** | 60 | 70 | negative |
| **S51** | NA | NA | NA |
| **S53** | 90 | 40 | negative |
| **S57** | 98 | 1 | negative |
| **S58** | 90 | 0 | negative |
