# Supplementary material for: ESR1 Gene Mutation in Hormone Receptor-Positive HER2-Negative Metastatic Breast Cancer Patients: Concordance Between Tumor Tissue and Circulating Tumor DNA Analysis
Source: Front Oncol. 2021 Mar 11;11:625636. doi: 10.3389/fonc.2021.625636 (PMC7991720; doi:10.3389/fonc.2021.625636)
Supplement: Supplementary file 4 [file Table_4.docx]

|  | **Site of metastasis** | | | | | | |
| --- | --- | --- | --- | --- | --- | --- | --- |
|  | **Liver (yes/no)** | **Lung (yes/no)** | **Other (yes/no)*** | **Lymphnodes (yes/no)** | **Bone (yes/no)** | **Brain (yes/no)** |  |
| **Patient** |  |  |  |  |  |  |  |
| **S01** | yes | no | yes | yes | yes | yes |  |
| **S02** | yes | no | no | yes | yes | no |  |
| **S03** | no | yes | no | yes | yes | no |  |
| **S06** | yes | yes | no | yes | yes | no |  |
| **S08** | yes | no | yes | yes | yes | yes |  |
| **S09** | yes | yes | yes | yes | yes | no |  |
| **S10** | yes | no | no | no | yes | no |  |
| **S11** | yes | no | no | yes | yes | no |  |
| **S12** | yes | no | yes | yes | no | no |  |
| **S13** | no | no | yes | no | no | no |  |
| **S14** | no | yes | yes | no | no | no |  |
| **S15** | yes | no | no | no | yes | no |  |
| **S16** | yes | yes | no | no | yes | no |  |
| **S17** | yes | yes | no | no | yes | no |  |
| **S18** | no | no | no | no | yes | no |  |
| **S19** | yes | no | no | no | yes | yes |  |
| **S20** | yes | no | no | no | yes | no |  |
| **S21** | yes | yes | yes | yes | yes | no |  |
| **S22** | no | no | no | yes | yes | no |  |
| **S24** | yes | no | no | no | no | no |  |
| **S25** | yes | no | no | no | yes | yes |  |
| **S26** | no | yes | no | yes | yes | no |  |
| **S27** | yes | no | no | no | yes | no |  |
| **S28** | no | no | yes | yes | yes | no |  |
| **S30** | yes | yes | no | no | no | no |  |
| **S31** | no | no | no | no | yes | no |  |
| **S32** | no | no | no | yes | yes | no |  |
| **S34** | no | no | no | no | yes | no |  |
| **S35** | yes | no | no | no | no | no |  |
| **S36** | no | no | no | no | yes | no |  |
| **S37** | no | no | no | no | yes | no |  |
| **S38** | yes | yes | no | no | yes | no |  |
| **S39** | yes | no | no | no | yes | no |  |
| **S40** | yes | no | no | yes | yes | yes |  |
| **S41** | no | yes | no | yes | yes | no |  |
| **S42** | no | no | no | yes | no | no |  |
| **S43** | yes | no | no | yes | yes | no |  |
| **S46** | no | no | no | yes | yes | no |  |
| **S49** | no | no | yes | no | no | no |  |
| **S51** | no | no | no | yes | no | no |  |
| **S53** | no | no | yes | yes | yes | no |  |
| **S57** | yes | yes | no | yes | yes | no |  |
| **S58** | no | no | no | no | yes | no |  |

**Supplementary Table 4**. Site of metastasis in the overall population.

*Other includes soft tissue, breast, skin.
