# Supplementary figures and images for: ESR1 Gene Mutation in Hormone Receptor-Positive HER2-Negative Metastatic Breast Cancer Patients: Concordance Between Tumor Tissue and Circulating Tumor DNA Analysis
Source: Front Oncol. 2021 Mar 11;11:625636. doi: 10.3389/fonc.2021.625636 (PMC7991720; doi:10.3389/fonc.2021.625636)

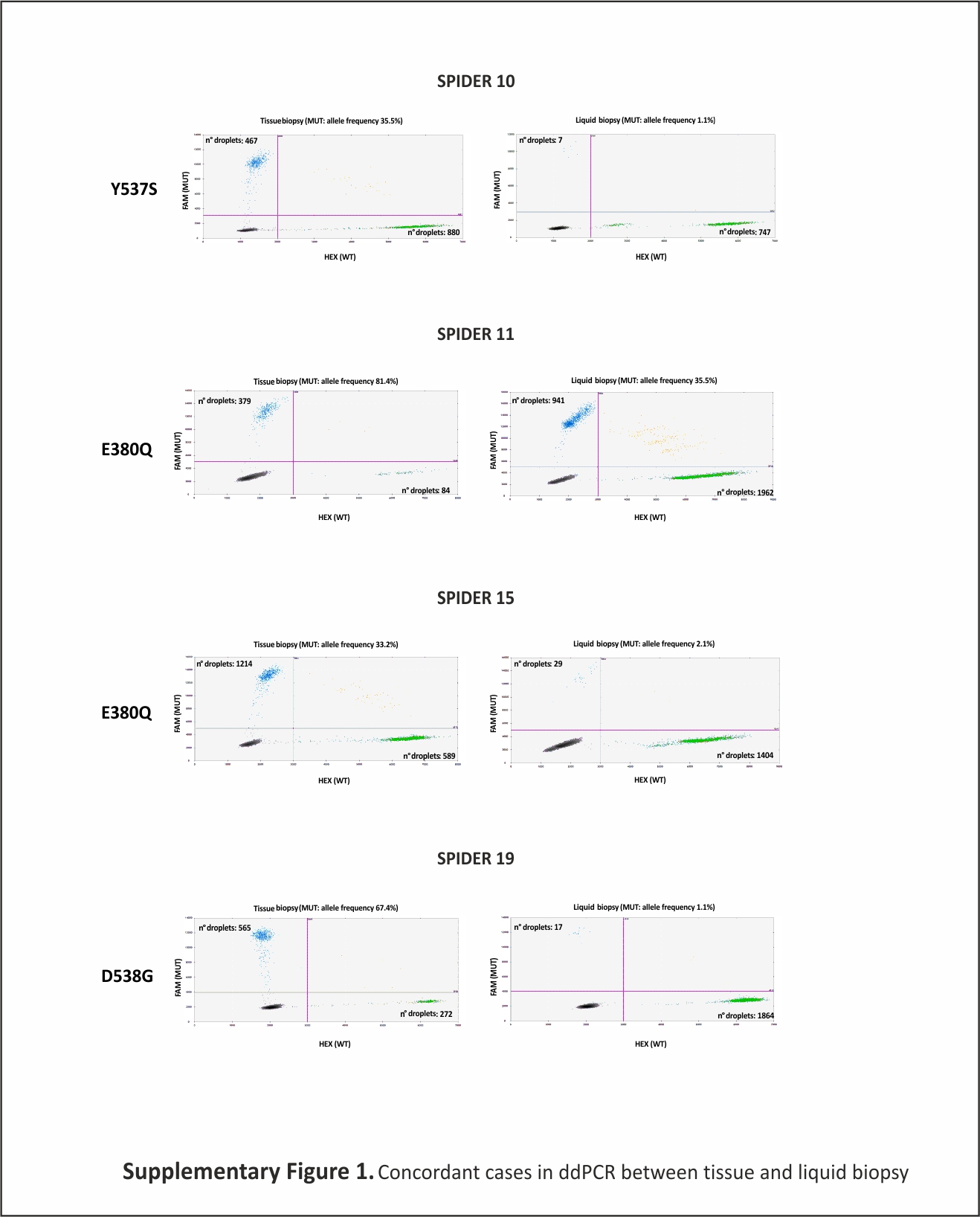

Supplement: Supplementary file 5 [file Image_1.jpeg]
